# Supplementary material for: Metastable phase-separated droplet generation and long-time DNA enrichment by laser-induced Soret effect
Source: Commun Chem. 2025 Feb 28;8:61. doi: 10.1038/s42004-025-01438-w (PMC11871339; doi:10.1038/s42004-025-01438-w)
Supplement: Supplementary file 2 — Supplementary Information [file 42004_2025_1438_MOESM2_ESM.pdf]

Supplementary Information for

Metastable phase-separated droplet generation

and long-time DNA enrichment by laser-induced Soret effect

Mika Kobayashi*, Yoshihiro Minagawa, and Hiroyuki Noji*

Department of Applied Chemistry, Graduate School of Engineering University of Tokyo,

Tokyo 113-8656, Japan

*Corresponding author.
E-mail address:
Mika Kobayashi: kobayashi.phys@gmail.com
Hiroyuki Noji: hnoji@g.ecc.u-tokyo.ac.jp

Contents

Supplementary Figures 1 to 5

Supplementary Note 1: Temperature increase by the laser irradiation

Supplementary Note 2: Case without ITO coating

Supplementary Note 3: Heating of a LIPS droplet

Supplementary Note 4: Estimation of DNA enrichment factor and Dex concentration ratio

Supplementary Note 5: Validity of the temporal change of the intensity ratio

Supplementary Note 6: DNA enrichment factor and DEX concentration ratio of droplets by. spontaneous phase separation

Supplementary Note 7: Localisation of DNA in a PEG solution

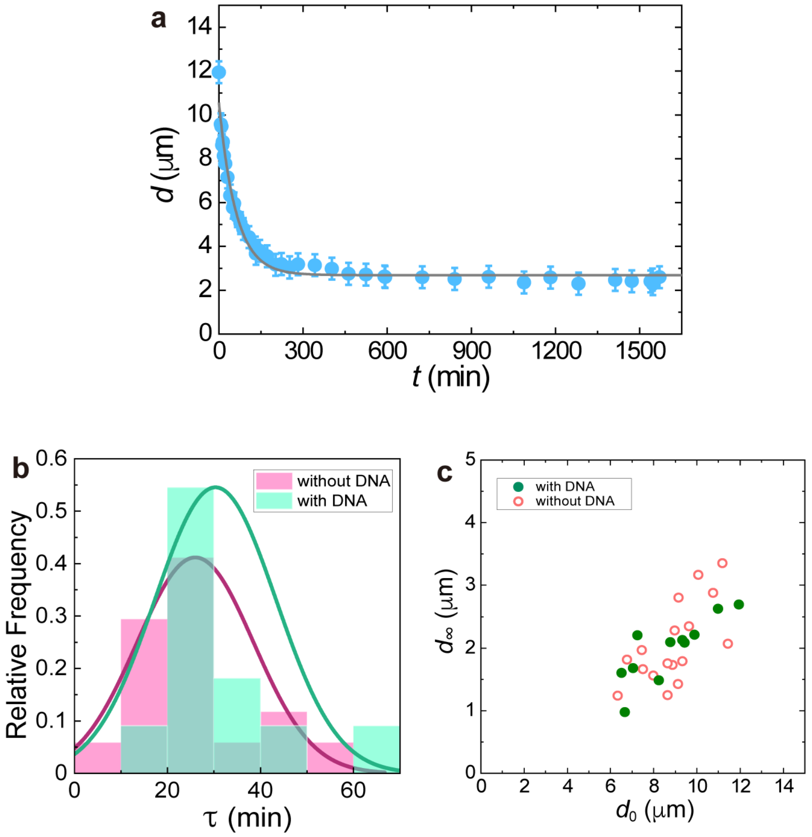

Supplementary Figure 1: Time evolution of droplet diameter. a, Time evolution of the droplet diameter for the same experiment shown in Fig. 4a-c in the main text. The droplet was generated by laser irradiation for 12 min to the upper PEG-rich phase of DEX 6 wt.% and PEG 2.6 wt.% + λ-DNA (48,502 bp) 29 ng/μL. The value at t = 0 was obtained from the phase-contrast image. The rest were obtained from the confocal images of DNA. The diameter approaches a finite value. The solid curve was obtained by fitting the data to a function, $d(t) = (d_0 - d_\infty)\exp(-t/\tau) + d_\infty$, where d_0 and d_∞ represent the diameters at $t = 0$ and $t = \infty$, respectively, and τ represents the characteristic decay time. The fitting parameters from the fit were τ = 60.7 min and d_∞= 2.7 μm. **b**, Histogram of the decay time of droplet diameter in the case with and without DNA. **c**, Final droplet diameter d_∞ at $t = \infty$ estimated by the fit plotted against the initial droplet diameter d_0 at $t = 0$ (measured from

phase contrast image). Green circles: cases with DNA. Red open circles: without DNA. The droplets were generated from the upper PEG-rich phase of DEX 6 wt.% and PEG 2.6 wt.% with/without DNA (λ-DNA (48,502 bp) 29 ng/μL)).

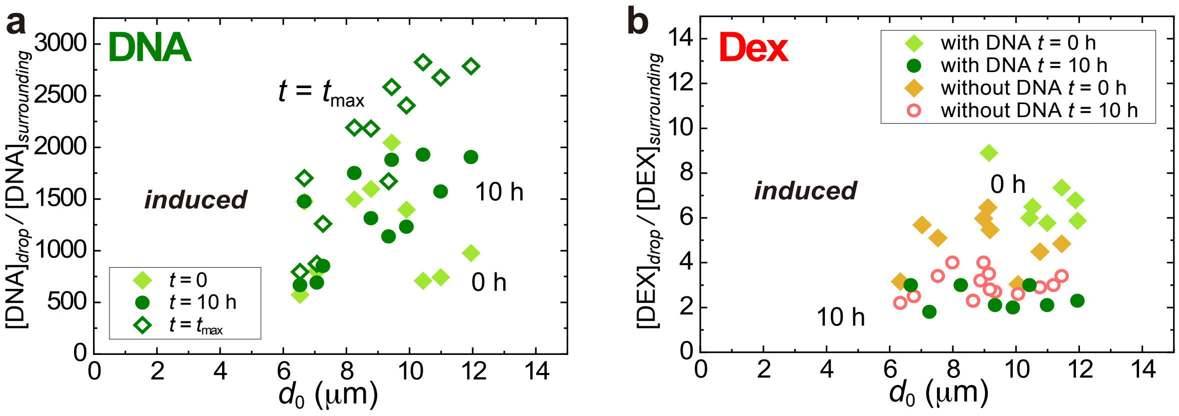

Supplementary Figure 2: The DNA enrichment factor and DEX concentration ratio. a, The DNA enrichment factor of LIPS droplets. The initial value (t = 0 h), maximum value ($t = t_{max}$), and long-time value (t = 10 h). The droplets were generated from the upper PEG-rich phase of DEX 6 wt.% and PEG 2.6 wt.% with DNA (λ-DNA (48,502 bp) 29 ng/μL). **b**, Concentration ratio of DEX between the droplet and surrounding phase of the induced droplets at t = 0, 10 h estimated from the fluorescence intensity ratio of dextran. The droplets were generated from the upper PEG-rich phase with/without DNA (DEX 6 wt.% and PEG 2.6 wt.% with/without DNA (λ-DNA (48,502 bp) 29 ng/μL).

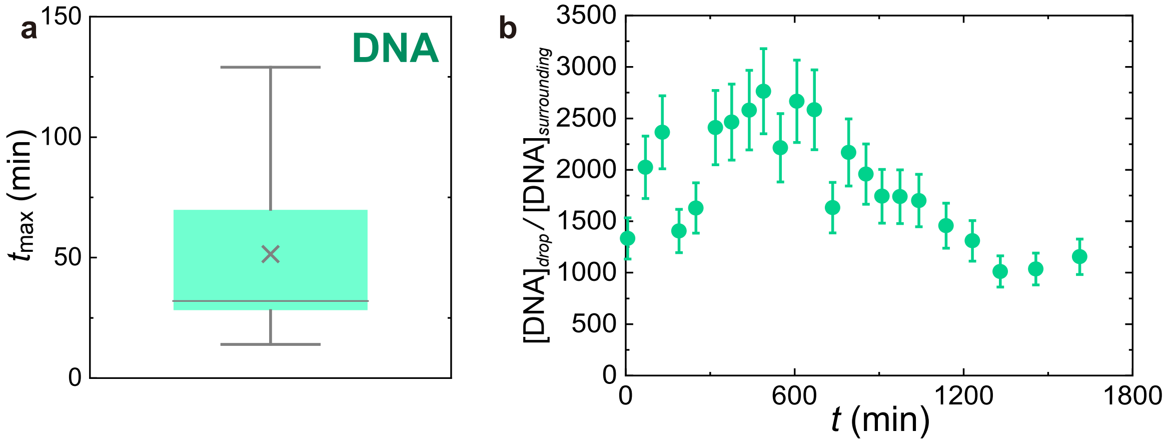

Supplementary Figure 3: Behaviour of DNA enrichment factor to reach its maximum value. a, Time scale when the enrichment factor of DNA (EF^{DNA}) reached its maximum value. 25–75 percentile (box), median (line), mean (cross), highest/lowest observations without outliers (whiskers). **b,** A case where EF^{DNA} showed a broad time change and showed its maximum value at a very long time around 500 min. The factor EF^{DNA} was kept at a high value of more than 2500 for an exceedingly long time.

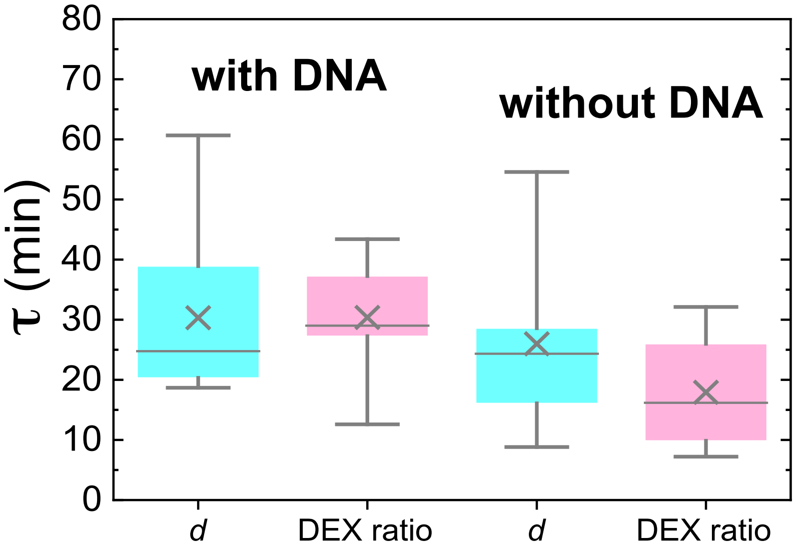

Supplementary Figure 4: Comparison of timescale between the droplet shrinkage and the DEX concentration change. Decay time was estimated by exponential fit. The function used for the fit was the same expression as that given in Supplementary Figure 1. Decay time of droplet diameter (light blue) and DEX concentration ratio (pink) in the case of w/ and w/o DNA. 25–75 percentile (box), median (line), mean (cross), highest/lowest observations (whiskers).

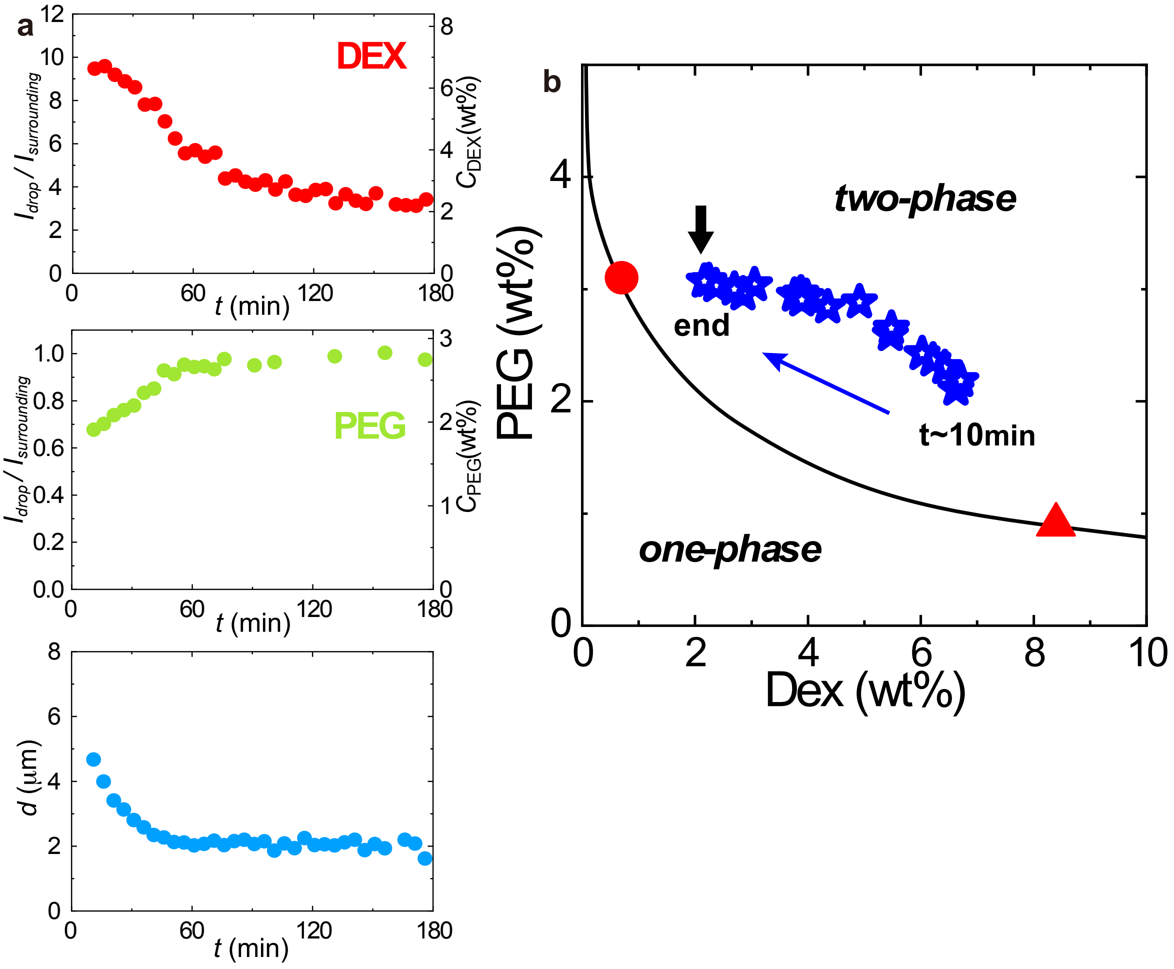

Supplementary Figure 5: An actual example of the composition change of LIPS droplets after the laser is switched off. **a**, Time course of the concentration ratio (= fluorescent intensity ratio) of DEX (top), the concentration ratio (= intensity ratio) of PEG (middle), and droplet diameter (bottom). Each concentration ratio was estimated from the intensity ratio of the average intensity inside droplet I_{drop} to that of the surrounding phase $I_{surrounding}$ (See Supplementary Note 4). The right axis in the figures (top and middle) denotes absolute concentration converted from the intensity ratio by multiplying the concentration of the surrounding phase. The droplet was generated by laser irradiation for 20 min to the upper PEG-rich phase of DEX 6 wt.% and

PEG 2.6 wt.% (DEX 5.8 wt.%, FITC-DEX 0.2 wt.%, PEG 2.4%, TRITC-PEG 0.2 wt.%, See Method in the main text). **b**, The time change of composition in the phase diagram for the data shown in Supplementary Figure 5a. A black arrow denotes the final composition of the droplet at *t* ~180 min, where the droplet seems to be in a metastable state.

Supplementary Note 1: Temperature increase by the laser irradiation

We estimated the temperature increase attributed to laser irradiation from the temperature dependence of fluorescence intensity. The temperature dependence of fluorescence intensity of 0.01 wt.% aqueous solution of Rhodamine B is illustrated in Supplementary Figure 6a. The intensity was normalised by that at 25 °C, which was the temperature in the droplet experiment. The intensity monotonically decreased with an increase in the temperature. Supplementary Figure 6b shows the intensity profile of a fluorescent image of the 0.01 wt.% Rhodamine B aqueous solution in a sample cell with ITO coating, which is captured after laser irradiation for 1 min. The intensity is normalised by the image captured before irradiation. The intensity at the laser-irradiation spot decreased from 1 to 0.85. The increase in temperature caused by laser irradiation was estimated at approximately 10 K. Changes in concentration attributed to the Soret effect of Rhodamine B are negligible (in the order of 10^{-5} wt.%/K).

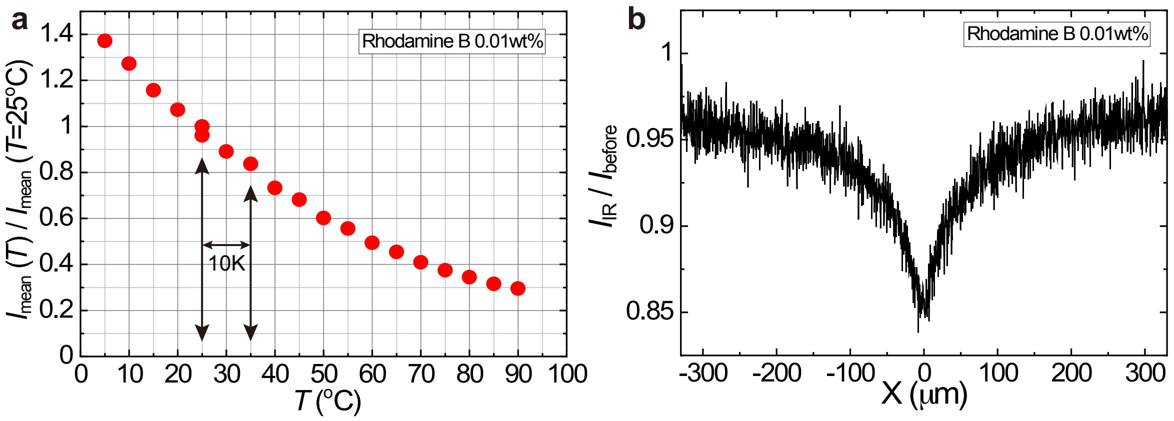

Supplementary Figure 6: Estimation of temperature increase. a, Temperature dependence of 0.01wt.% aqueous solution of Rhodamine B. **b,** Intensity profile of the fluorescent image of the sample after laser irradiation.

Supplementary Note 2: Case without ITO coating

We experimentally verified that our droplets were generated by the Soret effect by comparing two cases, i.e. with and without ITO coating (Supplementary Figure 7). When laser light is irradiated on the region without an ITO coating, no temperature gradient is produced. In this case, we did not identify any sign of a concentration change, which means the interaction between the sample and laser light does not induce phase separation in the sample and that local heating is essential for generating droplets.

Further, heating or cooling the bulk sample by a temperature stage in the range of 283–368 K did not cause anything to indicate a phase separation, as shown in Supplementary Figure 8. This confirms that the phase diagram of our system is inert to temperature. In other words, droplets cannot form without 'local' heating. Hence, the temperature gradient caused by local heating changes the local concentration due to the Soret effect, which results in phase separation.

The generation process (Fig. 1d in the main text) appears similar to that observed by Walton and Wynne[1,2]; however, the mechanisms are different. They estimated that the effect of thermophoresis is negligible in their system and concluded that their LIPS was caused by the electromagnetic energy stored in the sample by laser irradiation. In our case, the Soret effect causes droplet generation.

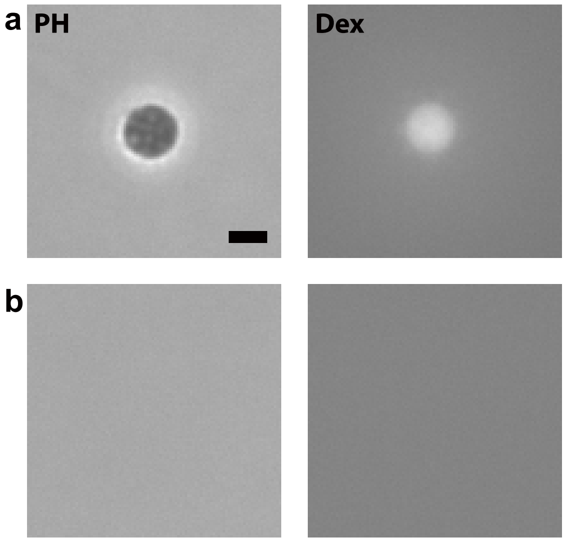

Supplementary Figure 7: Comparison of two cases with/without ITO coating. The droplet was induced by laser irradiation for 10 min to the upper PEG-rich phase of DEX 6 wt.% PEG 2.6wt.%. The same sample cell was irradiated with laser light in different regions (a) with and (b) without ITO coating. Left: phase-contrast images, right: fluorescent images of dextran. Scale bar = 5 μm.

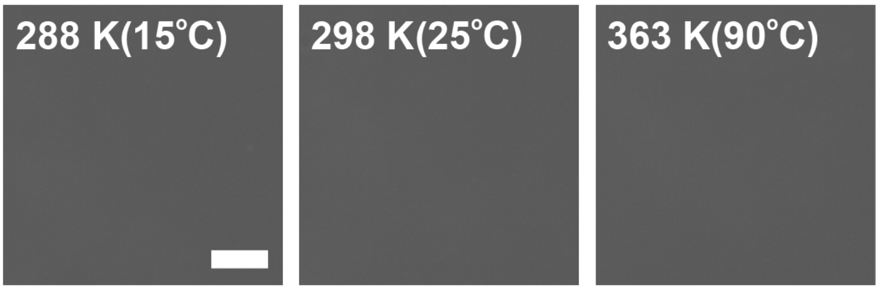

Supplementary Figure 8: Temperature dependence of a bulk sample. Phase contrast images of the upper PEG-rich phase of DEX 6 wt.% PEG 2.6wt.%. Scale bar = 20 μm.

Supplementary Note 3: Heating of a LIPS droplet

We examined if we can change the temperature of our LIPS droplet and it can be kept. We heated a LIPS droplet after switching off the laser, and the phase contrast images are shown in Supplementary Figure 9. The droplet is kept on heating or cooling. A decrease in the image contrast at high temperatures does not mean that the droplet is disappearing. The low contrast could be due to decreasing the difference in the refractive index between the droplet and the surrounding phase. The contrast returned to the original one when it was cooled again. This means that the droplet was stable on temperature change.

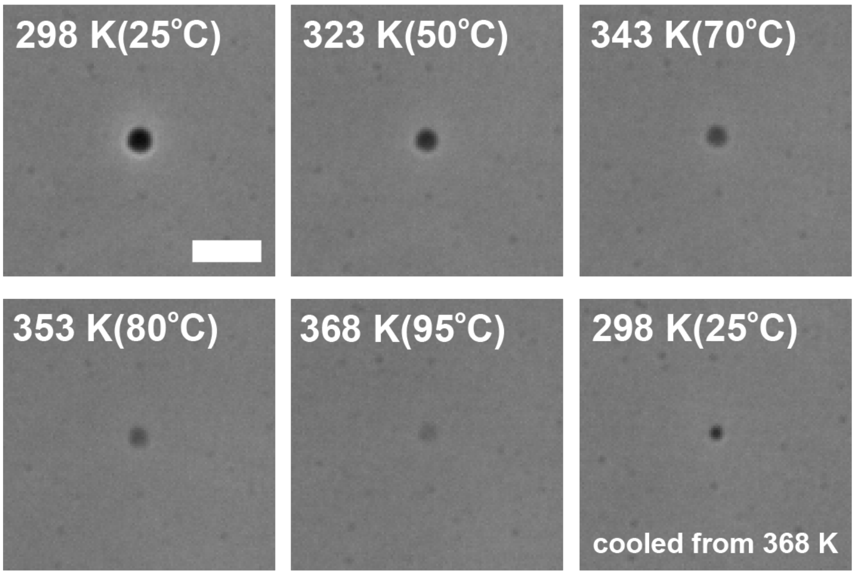

Supplementary Figure 9: Droplet image on heating. The droplet was first induced by laser irradiation for 5 min to the upper PEG-rich phase of DEX 6 wt.% PEG 2.6wt.% at 298 K. Phase-contrast images were taken when the whole sample cell was heated after generation. The image contrast of the droplet decreased on heating but returned on cooling to 298 K. Scale bar = 10 μm.

Supplementary Note 4: Estimation of DNA enrichment factor and Dex concentration ratio

1. DNA enrichment factor

We evaluated the DNA concentration of droplet $[DNA]_{droplet}$ and that of the surrounding phase (the upper PEG-rich phase) $[DNA]_{surrounding}$ separately, and we estimated the DNA enrichment factor as the concentration ratio $[DNA]_{droplet}$ / $[DNA]_{surrounding}$.

In the initial preparation of the DEX/PEG mix with DNA, we used 29 ng/μL of DNA as the concentration for the total mixture. However, most of it goes to the lower DEX-rich phase because DNA is enriched to the DEX-rich phase. Thus, the DNA concentration of the upper PEG-rich phase is very low (< 1 ng/μL). The concentration of nucleic acid below a few ng/μL is difficult to estimate by conventional methods such as UV spectroscopy. In our confocal experiments, the average intensity of fluorescence from SYBR gold is almost the same signal level as the one from pure water, implying that it is difficult to estimate accurate DNA concentration from the average intensity of fluorescent images. However, DNA molecules are visible because the full length of λ-DNA (48,502 bp) is about 16 μm. Thus, we estimated the low DNA concentration of the surrounding phase (upper PEG-rich phase) from the number of DNA molecules in a unit area of confocal images for calculating the enrichment factor.

For a low concentration range below 1 ng/μL, we observed DNA suspension in water by confocal microscopy at several DNA concentrations. The typical images are shown in Fig. 8a in the main text.

We analysed 10 images (image size: 155 μm × 155 μm) at each concentration and counted the number of DNA molecules in a unit area using commercial software (Image Pro Plus, Media Cybernetics). The spatial resolution of confocal measurements in the vertical (Z) direction was 193 nm (objective lens 100 X oil, N.A. 1.47), and the overlap of molecules in the Z-direction can be neglected. The DNA molecules were well isolated, and the analysis was successful below 600 pg/μL. The results are illustrated in Fig. 8b in the main text. The concentration dependence of the number of DNA molecules showed a good linearity in the concentration range of 3–600 pg/μL. We performed a linear fit to the concentration dependence and the result was used to estimate the DNA concentration of the upper PEG-rich phase used for LIPS experiments. The confocal images of the upper PEG-rich phase were analysed and estimated as $[DNA]_{surrounding} = 70 \pm 10$ pg/μL. A confocal image of the upper phase is shown in Fig. 8c in the main text.

For the high concentration range above 1 ng/μL, we evaluated the DNA concentration from the absolute fluorescent intensity of confocal images. We observed DNA suspension in water in the same cell used in the LIPS experiments and measured the average intensity of the image at the same Z-position of droplet generation close to the ITO surface. The result is shown in Supplementary Figure 10. The linearity is maintained in the concentration range of 1–1000 ng/μL. We increased the amount of SYBR gold concentrate at high concentrations above 100 ng/μL to maintain a sufficient amount of SYBR gold for DNA molecules. For the suspension of 100 ng/μL, the intensity was measured in

both conditions at 1/1000 and 1/100 of SYBR gold concentrate and the intensities were consistent with each other. In the LIPS experiment, we prepared all samples with 1/1000 of SYBR gold concentrate. The amount of SYBR gold is sufficient because the total DNA concentration for the initial mixture is 29 ng/μL, which is in the linear regime in the concentration dependence shown in Supplementary Figure 10. In addition, the initial DNA concentration of the PEG-rich phase was approximately 70 pg/μL, as shown above. The volume of one droplet was estimated to be in the order of 10 μm^3. This value is significantly smaller than the total sample volume by a factor of 10^{-8}. We concluded that the sample contains an adequate amount of DNA molecules and SYBR gold to achieve high concentration in the droplet, and that linearity is maintained under the experimental conditions of this study.

Supplementary Figure 11a shows an example of the time evolution of the absolute intensity of the LIPS droplet obtained as raw data. We converted the absolute intensity to DNA concentration (Supplementary Figure 11b). The enrichment factor was obtained by dividing the DNA concentration of the droplet by that of the surrounding phase (upper PEG-rich phase), i.e. $[DNA]_{droplet}$ /$[DNA]_{surrounding}$ (Supplementary Figure 11c). The experimental error of the enrichment factor was attributed to the estimation of the concentration value of the surrounding phase (upper PEG-rich phase) and the focal position in the confocal measurements. We considered these factors and determined the error bars in Fig. 4b in the main text as 15%.

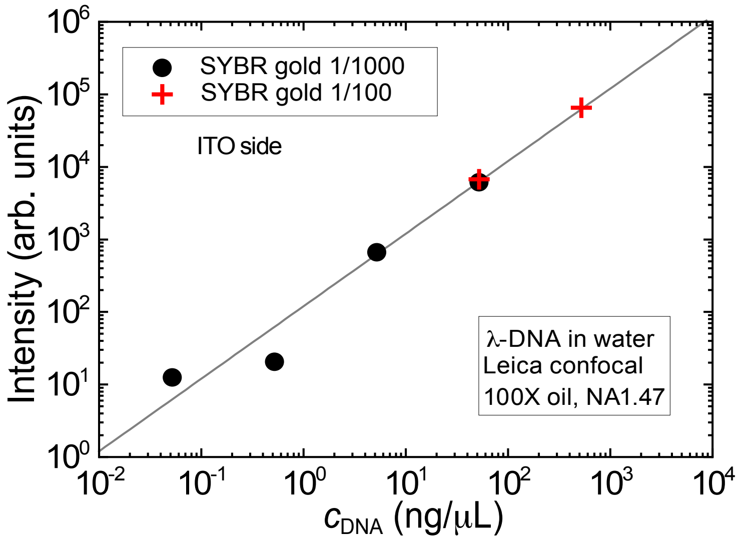

Supplementary Figure 10: DNA concentration dependence of fluorescence intensity from SYBR gold.

The fluorescence intensity of DNA suspension in pure water was measured in confocal microscopy at two conditions, 1/1000 (black circles), and 1/100 (red crosses) of SYBR gold concentrate. Experiments were performed at room temperature (297 ± 0.5 K).

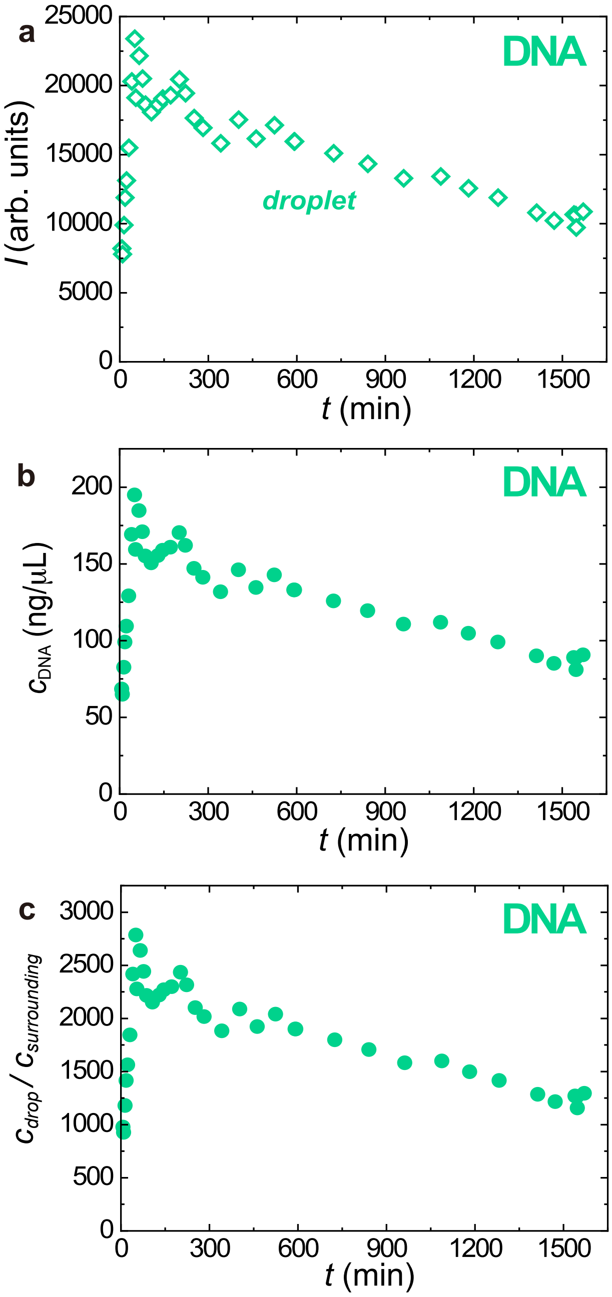

Supplementary Figure 11: Evaluation process of the DNA enrichment factor from confocal data.

a, raw data. **b,** absolute concentration. **c,** concentration ratio.

2. DEX concentration ratio

The DEX concentration ratio was estimated as the intensity ratio of the average intensity inside droplet I_{drop} to that of the surrounding phase $I_{surrounding}$, i.e. $I_{drop}/I_{surrounding}$. Before the estimation of the intensity ratio, the background signal in confocal measurements including the dark current of the camera, stray light, and fluorescent signal from substances excluding the target must be subtracted from raw data. The background intensity was measured as the average intensity of pure water in the same sample cell. In time-lapse measurements, a confocal image was obtained by averaging an appropriate minimum number of images for an adequate S/N of 1–16 frames. The average intensity of an image for the emission range to detect DEX is illustrated in Supplementary Figure 12a as a function of the number of images for averaging. The intensity is independent of the number of images for averaging.

To calculate $I_{surrounding}$, small ROIs (rectangular regions of approximately 30 μm on each side) were selected from several different positions (approximately 10) from an image, and subsequently, the average intensity and its standard deviation for each ROI were calculated. $I_{surrounding}$ was obtained as the average intensity of those ROIs. The raw data of I_{drop} and $I_{surrounding}$ in a time-lapse measurement are illustrated in Supplementary Figure 12b. The background intensity was subtracted from the raw data before calculating the intensity ratio. Finally, the DEX concentration ratio is calculated as shown in Supplementary Figure 12c. The experimental error of the intensity ratio was determined by the

possible error due to the focal position in the confocal measurements as the factor ± 0.6.

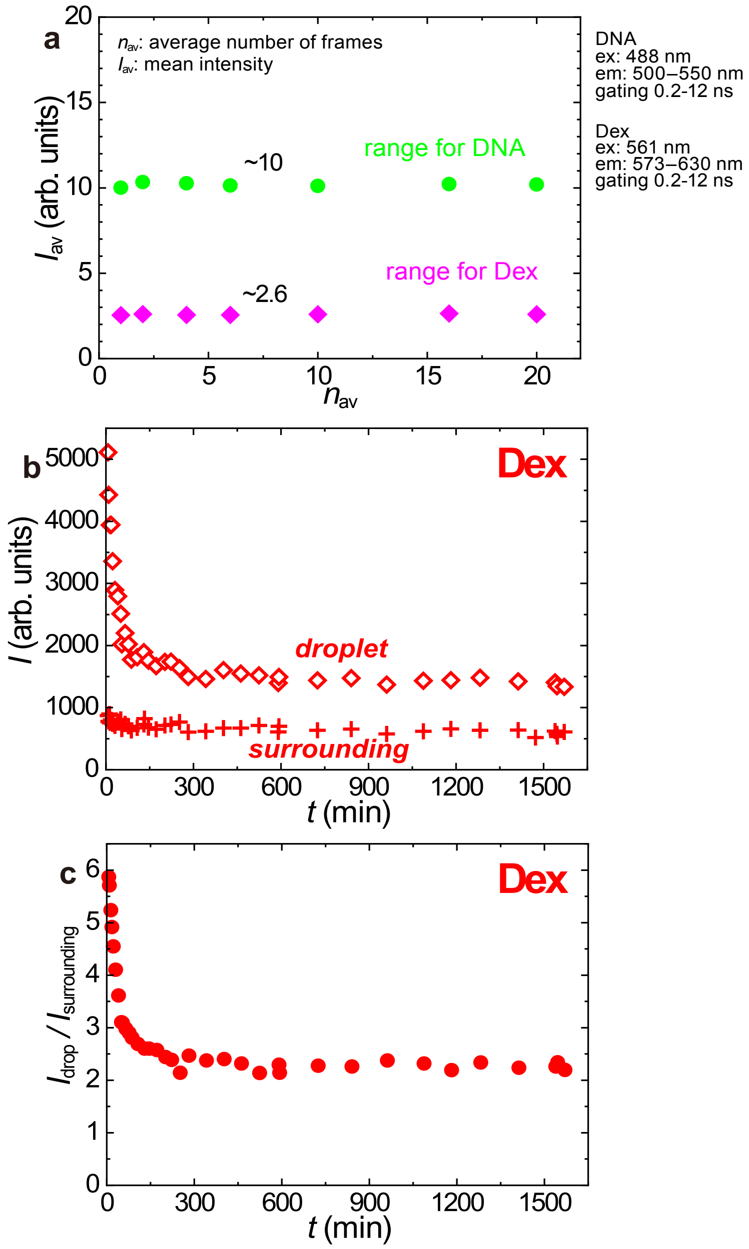

Supplementary Figure 12: Evaluation process of the DEX concentration ratio from confocal data.

a, background signal. **b,** raw data. **c,** intensity ratio.

Supplementary Note 5: Validity of the temporal change of the intensity ratio

We checked the possibilities affecting the temporal dependence to confirm that a decrease in the intensity ratio arises from concentration change.

The first possibility is the intensity decrease caused by photobleaching. The contribution of the photobleaching effect to the fluorescence intensity was estimated by the continuous repeat acquisition of spontaneous droplets. These droplets were produced solely by agitating the system in the two-phase region, and therefore, in principle, the DEX concentration inside the droplet should be constant with time. Supplementary Figure 13a illustrates the average intensity of 139 droplets by repeating the image acquisition 100 times. One round of acquisition took approximately 10 s. The intensity was normalised by the intensity of the first image, and we determined that photobleaching decreased intensity by less than 5 %. In the confocal measurement of the laser-induced droplet, an excitation laser irradiated the sample for 1–20 s for each image. A maximum of 50 timelapse images were obtained. The possible intensity decrease caused by photobleaching in the timelapse measurement was estimated at less than 5 %. The possible photobleaching effect for the DNA is also estimated to be less than 5 % from Supplementary Figure 13b.

Next, the signal leakage in multi-wavelength measurement was estimated. Supplementary Figure 14a illustrates the confocal image of DEX-rich droplets observed in the emission range used for detecting DEX (left) and DNA (right), where the intensity in the right-hand side figure is enhanced

by a factor of 25. This sample contained a fluorescent dye (TRITC-DEX 0.2 wt.%) only for DEX without DNA (DEX 6 wt.% (including TRITC-DEX 0.2 wt.%) PEG 2.6wt.%). The right-hand side image highlights the signal leakage to the emission range of the DNA. The relationship between the fluorescence intensity in the emission range of DEX and DNA for the same droplet is presented in Supplementary Figure 14a (bottom). The estimated signal leakage from the fluorescent DEX was approximately 4%.

Supplementary Figure 14b illustrates the results of estimating the signal leakage of SYBR gold (1/1000 of SYBR gold concentrate) to the emission range of DEX, where we observe DEX-rich droplets that do not contain a fluorescent dye for DEX but only for DNA (SYBR gold) (DEX 6 wt.% (TRITC-DEX 0 wt.%) PEG 2.6 wt.% + λ-DNA (48,502 bp, 29 ng/μL) + 1/1000 of SYBR gold concentrate). The intensity in the left-hand side figure is enhanced by a factor of 1,000. In this case, we observed an intensity distribution; however, the signal leakage was proportional to the original signal. The leakage of the SYBR gold signal was estimated at 0.1%.

Supplementary Figure 14c and d are raw data of the average intensity of an induced droplet in the timelapse experiment illustrated in Fig. 4 in the main text plotted with the signal leakage of other fluorescent dye estimated from real data. We concluded that the signal leakage was insufficient to cause time dependence.

Finally, we checked if the decrease in the DEX intensity ratio could be attributed to the

inappropriate focal position in confocal microscopy. Supplementary Figure 15 is the side-view (the XZ-slice image) of an induced droplet. The time interval between the two images was 10 min. The diameter is sufficiently large compared to a pixel size, and therefore, the intensity change can be considered a concentration change. There is a clear decrease in the average intensity; i.e. it decreased by a factor of 0.8. From above, we concluded that the DEX concentration ratio of the induced droplets decreased with time.

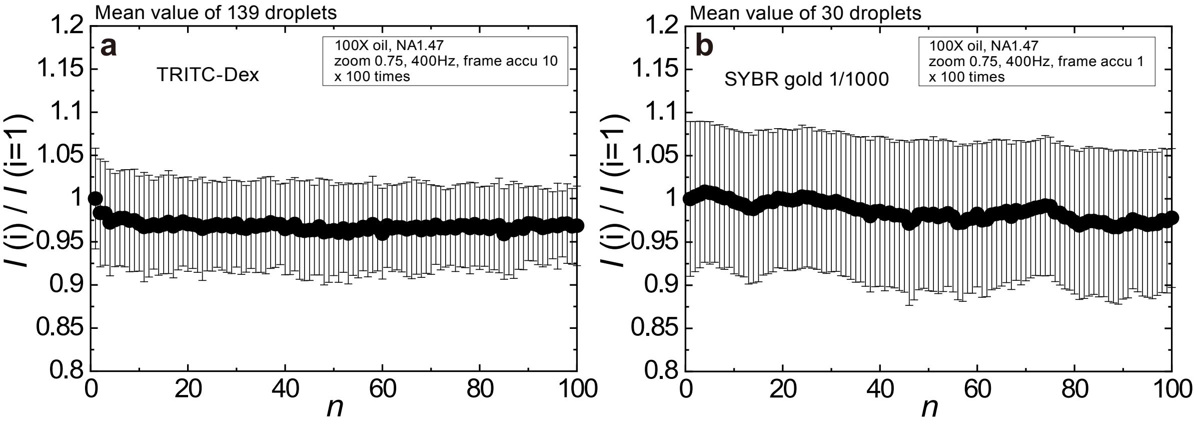

Supplementary Figure 13: Estimation of photobleaching. a, TRITC-DEX. **b,** SYBR gold.

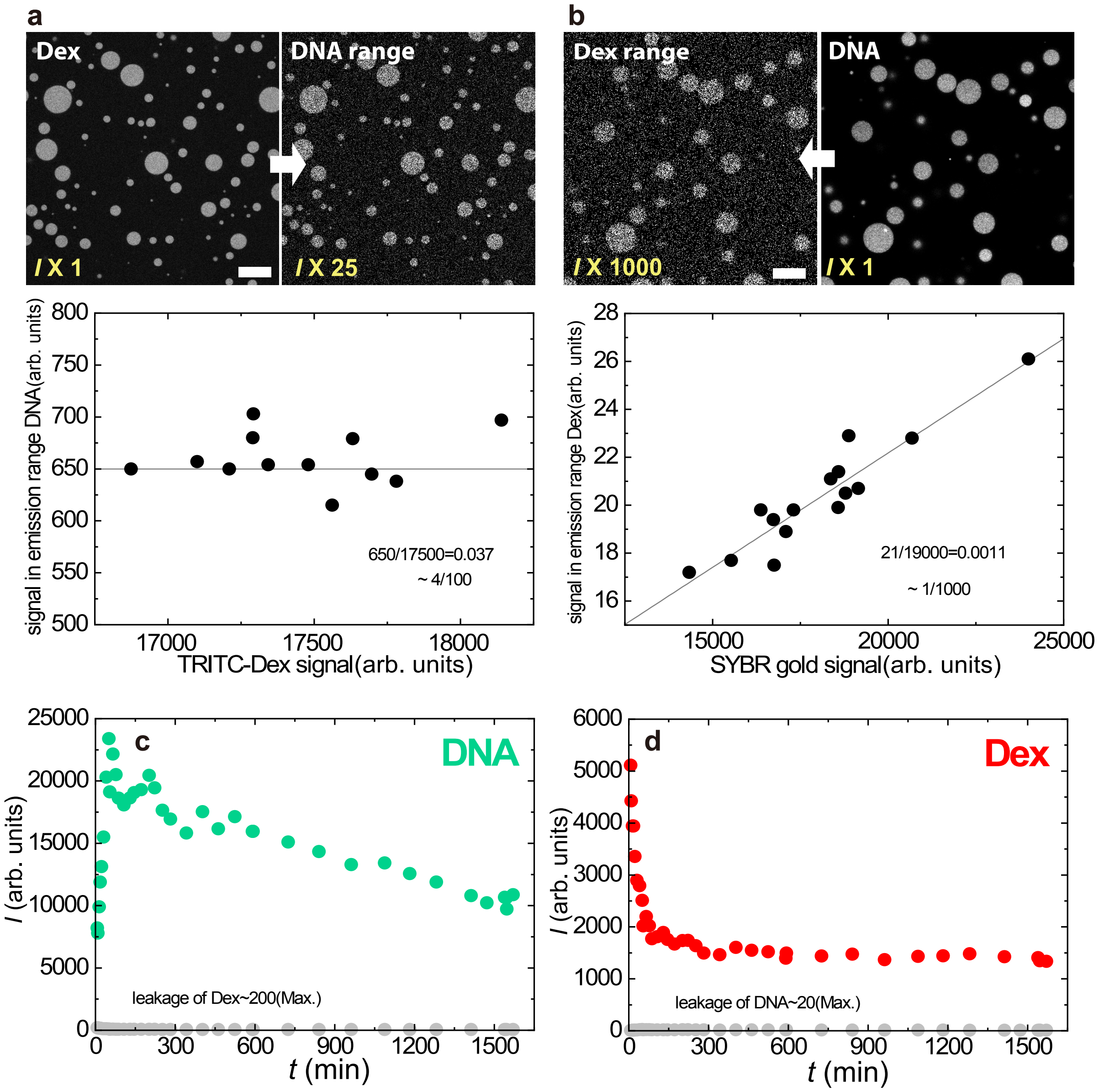

Supplementary Figure 14: Estimation of signal leakage in multi-wavelength detection. a, Confocal image of droplets visualised by TRITC-DEX (no SYBR gold). Scale bar = 20 μm. **b,** Droplets visualised by SYBR gold (no TRITC-DEX). Scale bar = 20 μm. **c,** Raw fluorescence intensity from DNA plotted with the estimated signal leakage from the DEX signal. d. Raw fluorescence intensity from DEX plotted with the estimated signal leakage from the DNA signal.

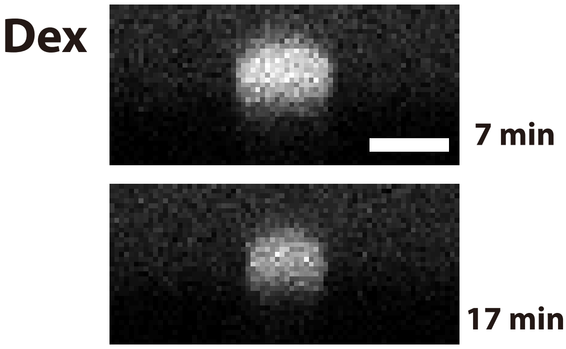

Supplementary Figure 15 : Side view of an induced droplet. The XZ-slice images of a droplet visualised by fluorescent DEX (TRITC-DEX). The droplet was induced by laser irradiation for 12 min to the upper PEG-rich phase of DEX 6 wt.% PEG 2.6wt.% + λ-DNA (48,502 bp, 29 ng/μL). (top) t = 7 min. (bottom) t = 17 min after switching off the laser. The intensity ratio between the droplet and the surrounding phase decreased from 7.5 (t = 7 min) to 6 (t = 17 min). Scale bar = 5 μm.

Supplementary Note 6: DNA enrichment factor and DEX concentration ratio of droplets by spontaneous phase separation

The DNA enrichment factor and DEX concentration ratio of droplets produced by spontaneous phase separation were estimated under the same condition of confocal microscopy as the laser-induced droplet generation experiments. We prepared a sample of DEX 6 wt.% and PEG 2.6wt.% + λ-DNA (48,502 bps, 29 ng/μL). Micrometre-size phase-separated droplets were formed by agitating the sample in a conventional vortex mixer. We awaited the sedimentation of droplets for approximately 30 min and extracted the upper PEG-rich phase containing an appropriate number of residual droplets to observe the isolated droplets under a microscope's field of view. For an appropriate comparison with the value obtained in the droplet induction experiment, we placed the sample in the same sample cell used for the droplet generation experiment and awaited the sedimentation of droplets to the ITO surface. Then, we observed the droplets contacted with the ITO surface under the same experimental condition. Typical images of the droplets in Supplementary Figure 16. The DNA enrichment factor and DEX concentration ratio were estimated by the same method as in Supplementary Note 4. The results are illustrated in Fig. 5 in the main text.

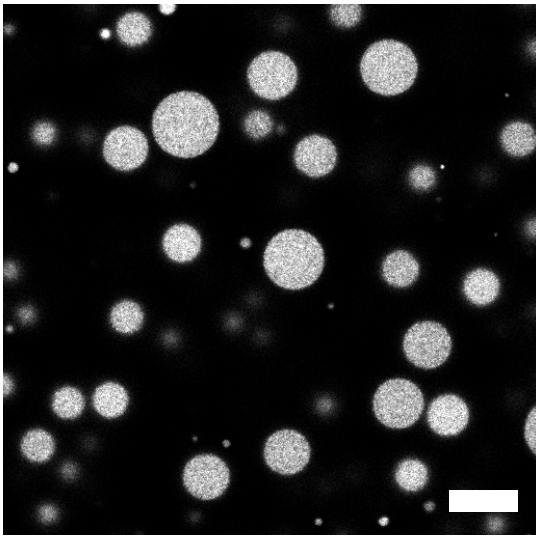

Supplementary Figure 16: Phase-separated DEX-rich droplets obtained by agitation. Droplets containing DNA were visualised using SYBR gold. DEX 6 wt.% and PEG 2.6wt.% + λ-DNA (48,502 bp, 29 ng/µL) at 1/1,000 of SYBR gold concentrate. Scale bar = 20 µm.

Supplementary Note 7: Localisation of DNA in a PEG solution

Duhr and Braun's[3] investigation into DNA suspension under a temperature gradient revealed that the DNA itself exhibits the Soret effect. They reported that the Soret coefficient S_T of the DNA changed its sign around 4 °C and $S_T > 0$ at room temperature (DNA moves away from the hot place). Maeda et al. revealed that, when DNA is added to a PEG solution, its behaviour depends on the length of the DNA and PEG concentrations[4,5]. At high concentrations of PEG, the DNA molecules can be localised to the hot region.

To compare the DNA enrichment in the induced phase-separated droplet with the localisation of DNA caused by the Soret effect itself[2,4,5], we performed local heating experiments in a DNA suspension in a PEG solution without DEX (PEG 3wt.% + DNA), which is approximately the same concentration as that of the system used in the droplet generation experiments (PEG 3.1 wt.%). The temporal evolution of fluorescent images that visualise DNA after laser irradiation for 5 min is illustrated in Supplementary Figure 17a. This system did not contain DEX, and it adhered to the condition that phase separation does not occur. Even without phase separation, the DNA molecules were localised at the irradiation spot by local heating at t = 0 s after switching off the laser. The intensity ratio in Supplementary Figure 17b was calculated by epifluorescence microscopy (not confocal); this ratio does not correspond to the enrichment factor. However, we can discuss the time scale of diffusion. Note that we performed similar experiments on both DNA in pure water and DNA

in the DEX solution at the same concentration as that of each component of the droplet generation experiments. In both cases, the DNA molecules moved away from the hot region created by laser irradiation (Supplementary Figure 18).

Further, we performed laser irradiation experiments on a sample of DNA suspension including DEX (DEX 1 wt% PEG 2.4 wt%) that is very close to the composition of the upper PEG-rich phase that is used in the LIPS experiments (DEX 0.7 wt%, PEG 3.1wt%). In that case, DNA formed a ring-shaped localisation. But the localisation disappeared in a few minutes (Supplementary Figure 19). The concentration increase of DEX due to the Soret effect can be seen, and the concentration distribution disappeared in a few min.

The localisation of DNA in the PEG solution is consistent with the observations in the literature[4,5]. However, the temporal evolution was entirely different from the enrichment in a phase-separated droplet. The diffusion of DNA was considerably fast. The intensity homogenized within minutes, which corresponded to a vanishing DNA localisation. These results suggest that the long-time localisation of DNA in the LIPS droplet induced by the Soret effect is not caused by the high concentration of PEG, and is instead caused by the generation of a phase-separated droplet.

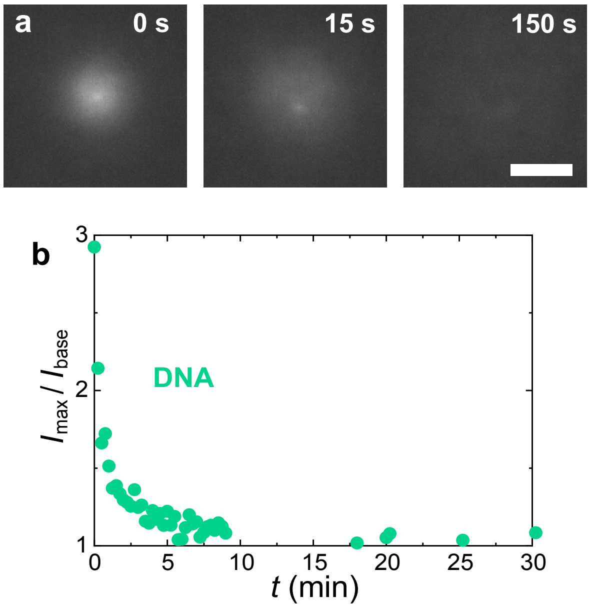

Supplementary Figure 17: Case of local heating in a DNA suspension in a PEG solution (3 wt.%). a, The temporal evolution in the fluorescent images of the DNA after the laser is disabled. PEG 3 wt.% + λ-DNA (48,502 bp, 1ng/μL). In this experiment, we used λ-DNA tagged with a fluorescent dye MFP-488 obtained with nucleic acid labelling reagents (Mirus Bio LLC, label IT, MFP-488). The laser irradiation time was 5 min. The time after the laser was switched off is indicated on the upper right-hand side of each image. DNA is localised at the laser-irradiated position; however, it diffuses rapidly in a few minutes when the laser is disabled. Scale bar = 5 μm. **b,** Temporal evolution of maximum fluorescence intensity normalised by the intensity of the surrounding region. A small bright spot in Supplementary Figure 17a is an aggregate or dust, which was excluded from the analysis. The time scale is considerably faster than in the case of DNA enrichment in the phase-separated droplet.

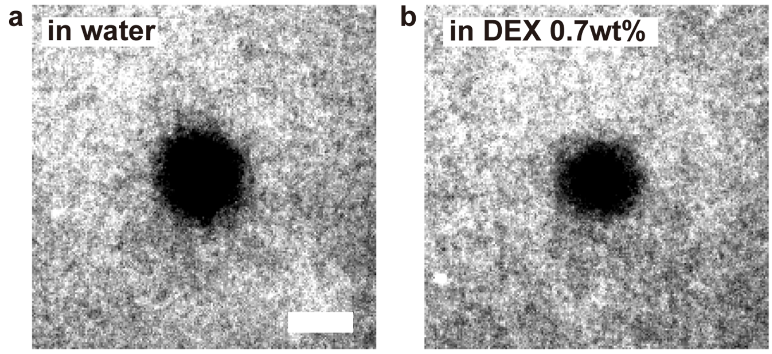

Supplementary Figure 18: Case of local heating in DNA suspensions. Fluorescent images of DNA visualised by SYBR gold were taken after the laser irradiation for 1 min. Scale bar = 50 μm. **a,** DNA suspension in water. λ-DNA (48,502 bp, 1.7ng/μL). **b,** DNA suspension in DEX 0.7 wt% solution. λ-DNA (48,502 bp, 1.7ng/μL).

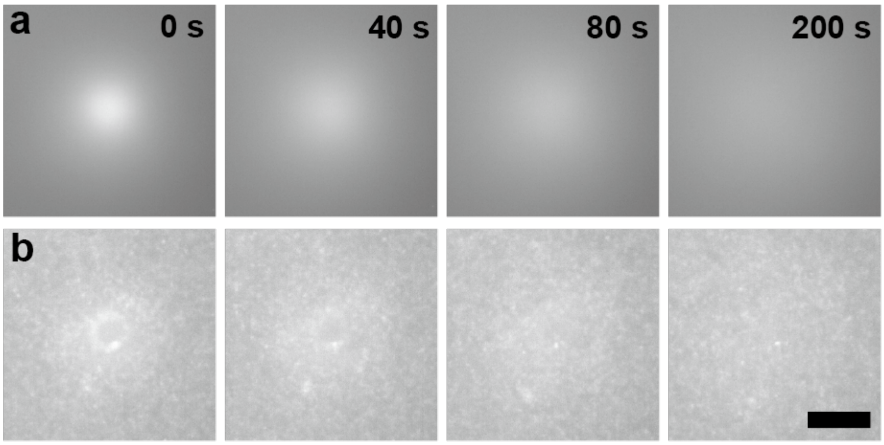

Supplementary Figure 19: Case of a sample in a single-phase region. Time evolution of the fluorescent image of a sample in one phase region (DEX 1 wt% PEG 2.4 wt% + λ-DNA (48,502 bp, 1.7ng/μL)) after laser irradiation for 5 min. **a**, DEX **b**, DNA at t = 0 s, 40 s, 80 s, 200 s from the left to the right.

Supplementary References

1. Walton, F. & Wynne, K. Control over phase separation and nucleation using a laser-tweezing potential. *Nat Chem* **10**, 506–510 (2018).

2. Walton, F. & Wynne, K. Using optical tweezing to control phase separation and nucleation near a liquid–liquid critical point. *Soft Matter* **15**, 8279–8289 (2019).

3. Duhr, S. & Braun, D. Why molecules move along a temperature gradient. *Proc National Acad Sci* **103**, 19678–19682 (2006).

4. Maeda, Y. T., Buguin, A. & Libchaber, A. Thermal Separation: Interplay between the Soret Effect and Entropic Force Gradient. *Phys Rev Lett* **107**, 038301 (2011).

5. Maeda, Y. T., Tlusty, T. & Libchaber, A. Effects of long DNA folding and small RNA stem–loop in thermophoresis. *Proc National Acad Sci* **109**, 17972–17977 (2012).
