# Supplementary material for: Metastable phase-separated droplet generation and long-time DNA enrichment by laser-induced Soret effect
Source: Commun Chem. 2025 Feb 28;8:61. doi: 10.1038/s42004-025-01438-w (PMC11871339; doi:10.1038/s42004-025-01438-w)
Supplement: Supplementary file 3 — Description of Additional Supplementary Files [file 42004_2025_1438_MOESM3_ESM.pdf]

Description of Additional Supplementary Files

Supplementary Movie 1: Droplet generation process by LIPS

The laser irradiation process for 10 min and the scenario after disabling the laser. This process was observed using phase contrast microscopy. Sample: upper phase of DEX (Mw550,000) 6 wt.% + PEG (Mw35,000) 2.6 wt.% + λ-DNA 29 ng/μL. A square with a side of 169 μm. Playback speed 10X.

Supplementary Movie 2: Heart shape patterning process of LIPS droplets

Patterning process of LIPS droplets in a heart shape. Sample: upper phase of DEX (Mw550,000) 6 wt.% + PEG (Mw35,000) 2.6 wt.% + λ-DNA 29 ng/μL. A square with a side of 676 μm. Playback speed 100X.

Supplementary Movie 3: Coalescence of two LIPS droplets

Two DEX droplets generated by LIPS were coalesced by manipulation. The process was observed by phase contrast microscopy. Sample: upper phase of DEX (Mw550,000) 6 wt.% + PEG (Mw35,000) 2.6 wt.%. A square with a side of 84.5 μm. Playback speed 1X.

Supplementary Movie 4: Laser-induced disappearance of LIPS droplet

The laser irradiation process to the LIPS droplet observed by phase contrast microscopy. The droplet disappeared after successive laser irradiation. The final disappearance depends on the balance between the mixing effect and the induction of new droplets by laser irradiation. Sample: upper phase of DEX (Mw550,000) 6 wt.% + PEG (Mw35,000) 2.6 wt.%. A square with a side of 33 μm. Playback speed 5X.

Supplementary Movie 5: Laser irradiation to a spontaneous droplet

The laser irradiation process to a spontaneous droplet observed by phase contrast microscopy. The droplet did not disappear after successive laser irradiation. The image contrast becomes low during the laser irradiation, but the droplet still appears after switching off the laser. Sample: upper phase of DEX (Mw550,000) 6 wt.% + PEG (Mw35,000) 2.6 wt.%. A square with a side of 33 μm. Playback speed 5X.
