# Supplementary material for: Metastable phase-separated droplet generation and long-time DNA enrichment by laser-induced Soret effect
Source: Commun Chem. 2025 Feb 28;8:61. doi: 10.1038/s42004-025-01438-w (PMC11871339; doi:10.1038/s42004-025-01438-w)
Supplement: Supplementary file 1 — Peer Review File [file 42004_2025_1438_MOESM1_ESM.pdf]

This manuscript has been previously reviewed at another journal. This document only contains information relating to versions considered at Communications Chemistry.

nature portfolio

Peer Review File

Metastable phase-separated droplet generation and long-time DNA enrichment by laser-induced Soret effect

Corresponding Authors: Dr Mika Kobayashi and Prof Hiroyuki Noji

Version 0:

Reviewer comments:

Reviewer #1

(Remarks to the Author)
The reviewer found that the authors improved the descriptions in correct, suitable matter, in response to the comments. Now, the reviewer recommends the acceptance of the revised manuscript for the publication.

Reviewer #2

(Remarks to the Author)
The authors have addressed my issues and I think it is now in a publishable form.

Open Access This Peer Review File is licensed under a Creative Commons Attribution 4.0 International License, which permits use, sharing, adaptation, distribution and reproduction in any medium or format, as long as you give appropriate credit to the original author(s) and the source, provide a link to the Creative Commons license, and indicate if changes were made.
In cases where reviewers are anonymous, credit should be given to 'Anonymous Referee' and the source.
The images or other third party material in this Peer Review File are included in the article’s Creative Commons license, unless indicated otherwise in a credit line to the material. If material is not included in the article’s Creative Commons license and your intended use is not permitted by statutory regulation or exceeds the permitted use, you will need to obtain permission directly from the copyright holder.
To view a copy of this license, visit https://creativecommons.org/licenses/by/4.0/
